# Assessing the Correlation between Skeletal and Corresponding Soft-Tissue Equivalents to Determine the Relationship between CBCT Skeletal/Dental Dimensions and 3D Radiographic Soft-Tissue Equivalents

**DOI:** 10.1155/2018/8926314

**Published:** 2018-07-03

**Authors:** Da In Kim, Manuel O. Lagravère

**Affiliations:** ^1^School of Dentistry, Faculty of Medicine and Dentistry, University of Alberta, ECHA, 11405-87 Avenue, Edmonton, AB, Canada T6G 1C9; ^2^School of Dentistry, Faculty of Medicine and Dentistry, University of Alberta, Edmonton, AB, Canada T6G 1C9

## Abstract

**Objective:**

Compare measurements of skeletal and dental areas on the CBCT to the corresponding soft-tissue measures taken from a 3D Facial Scanner.

**Methods:**

30 patients with CBCT and 3D Facial scanner photos were selected from the orthodontic program database. 30 different distance measurements were obtained from CBCT and facial scan. OrthoInsight software was used to obtain the measurements from the facial scan images, and AVIZO software was used for corresponding CBCT landmarks. The Euclidean distance formula was used to determine the distances for the corresponding *x*, *y*, and *z* coordinates of the CBCT. Reliability for CBCT and Facial Scanner was completed by calculating 30 distances for 10 patients, 3 times. Once reliability was determined, all 30 distances were calculated once for CBCT and facial scanner on each patient and descriptive statistics and paired *t*-test were applied.

**Results:**

All distances measured presented excellent reliability, the lowest one being the left eye width for the facial scanner (ICC 0.847). The landmark with the highest mean error on the CBCT was 2.0 ± 1.6 mm on the *z*-axis for the spinal level landmark. The Facial Scanner's largest mean measurement error was 1.5 ± 0.9 mm for the distance of the left corner of the mouth to gonion. All data except width between outer eye corners were statistically significant (*p* < 0.05). The average differences between facial scan and CBCT measurements ranged between 0.77 mm (left canine to cheekbone) to 26.94 mm (left subnasale to gonion) and are thus comparable. All measurements show a reasonable standard deviation between 2.57 mm (left eye width) to 9.91 mm (left gnathion to EAM).

**Conclusion:**

Distances obtained from CBCT and facial scan present mild differences giving the perspective of a relationship between them. Understanding this difference and relationship can make it plausible to expect certain underlying skeletal distances under soft-tissue structures.

## 1. Introduction

Since the development of dental photographic tools, much emphasis is put on skeletal landmarks as a tool for measurement in orthodontic analysis. In addition to skeletal evaluation, facial soft tissue evaluation plays a relevant role in treatment planning [1], since facial changes must be estimated while a patient undergoes long-term treatment. Both soft- and hard-tissue analyses as well as a more exact prediction of hard and soft tissue changes are important tools to help the clinician assess treatment outcomes and give added diagnostic information about the patient [2].

2D lateral cephalometric imaging has been the routine method of obtaining hard-tissue information of the patient [3]. In recent years, high precision of three-dimensional (3D) cone-beam computed tomography (CBCT) scanners and their clinically insignificant errors has gauged interest in many clinicians to use this as a routine tool for hard-tissue investigation during treatment planning and diagnosis [4]. However, 3D hard-tissue analysis alone is inadequate for proper treatment planning. Since soft-tissue profile reflects underlying skeletal tissue, visual inspection and examination of the patient can give insightful information of the underlying dental tissue [5]. Conventional methods for facial soft-tissue analysis include 2D measurement methods, such as taking photos of the patient at different angles [6]. These photos are then used to measure certain distances via computational analysis. Over the years, 3D facial soft-tissue analysis has been introduced to provide a more accurate description of the patient's soft-tissue profile [6]. These 3D facial scanners use a strip of laser light to record the contour of the patient's face and cranium and project their recordings onto a computer. With this 3D information, clinicians are able to obtain information such as cranial growth changes and treatment outcomes in a more realistic fashion. This ultimately allows the clinician to undergo prediction planning for the patient [7].

As such, both 3D soft- and hard-tissue analyses are essential in obtaining precise measurements for treatment planning. However, precise facial measurements can only be made when the clinician truly understands the relationship between these two imaging modalities and by obtaining a truthful 3D model of the soft tissues and underlying skeletal structures [7]. By determining relationships and assessing imaging tendencies between CBCT and facial scanner, clinicians will be able to deliver diagnoses with increased exactness: if the soft-tissue distance is highly correlated with that of the hard tissue, the clinician can conclude that this particular distance on the skin can highly reflect its underlying hard-tissue distance. Also, these would be the initial steps towards verifying the effects of treatments (orthodontic or surgical) on soft tissues when viewing in three-dimensions. The objective of this study is therefore, to analyze different landmark relations obtained from 3D facial scanner and CBCT for comparison and prediction planning, for use as a diagnostic tool.

## 2. Materials and Methods

CBCTs and 3D facial scans from 30 patients that were seen in the University of Alberta Graduate Orthodontic Clinic were selected for analysis. The basis for this sample size was based on availability of the images needed for the purpose of this study, since the CBCT and facial scan images were all taken retrospectively and were chosen amongst a main database. The reasoning of the full field of view CBCTs for these patients was for diagnostic and treatment planning purposes of the orthodontists in charge of the individual patient cases and was not taken for the purpose of this study. The University of Alberta's Human Research Ethics Board approved of this study (Pro00057947). CBCT scans of 0.3 mm voxel size were taken with the I-CAT Next generation device (9 sec exposure time, 13 cm *x* 16 cm FOV, 0.3 mm voxel size, Imaging Sciences International, Hatsfield, PA) at 120 kV, 5 mA with 8 mm aluminum filtration according to manufacturer's settings. 3D facial scans were obtained on the same day as the CBCTs, using Ortho Insight 3D Scanner (Motion View LLC., United States of America). All images were chosen with patients in their natural upright head position, with the Frankfort plane parallel to the floor. As all data were collected retrospectively, strict positioning of the head was not available to be controlled. CBCTs were analyzed using a third party software called AVIZO (Thermo Fisher Scientific, Hillsboro, United States of America), which helped to obtain the 3D reconstruction of the image for landmark positioning.

In relation to a reference point, each CBCT landmark (Tables 1 and 2) was given coordinates in *x*, *y*, and *z* format. This reference point was an arbitrary position placed amongst the coordinates of the software program. Since the distance between two specific points were to be measured, the initial reference point for each distance was different for each patient and distance, as it was all relative to where the second point was to be placed. The Euclidean distance formula was used to determine the linear distances for the corresponding *x*, *y*, and *z* coordinates.

(1) $$\begin{array}{c} d=\sqrt{{\left(x_{1}-x_{2}\right)}^{2}+{\left(y_{1}-y_{2}\right)}^{2}+{\left(z_{1}-z_{2}\right)}^{2}}. \end{array}$$

The facial scanning machine along with its corresponding third party software, OrthoInsight, was used to obtain the 3D soft-tissue profile of the patient. Soft-tissue distances between landmarks (Table 3) were calculated by the software to obtain landmark measurements in millimetres.

**Figure 1:**
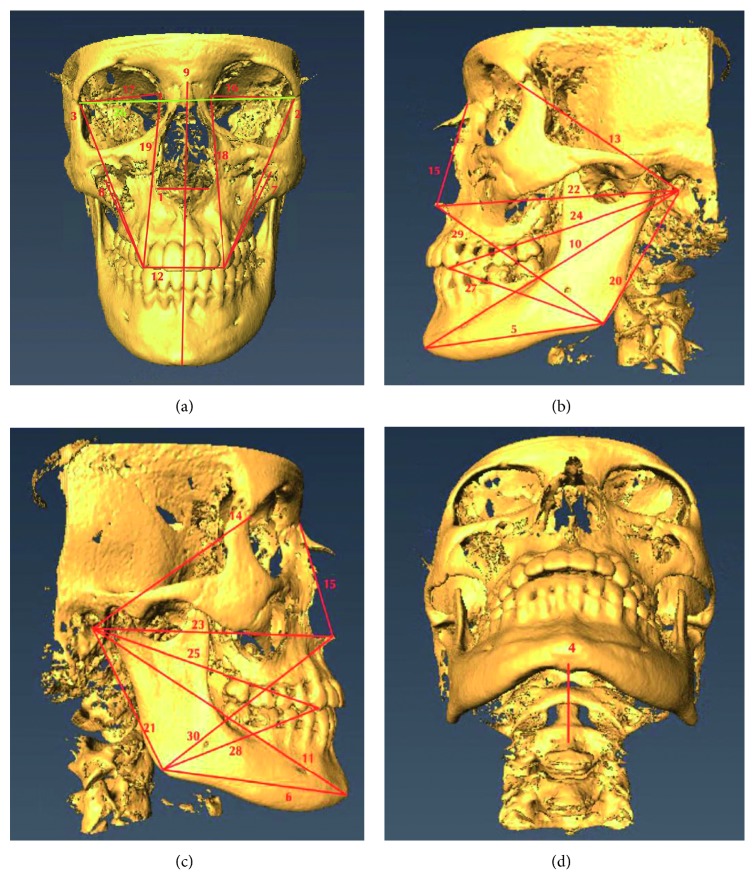
Front, left lateral profile, right lateral profile, and inferior views of the CBCT image, with numbered areas corresponding to landmarks. (a) Frontal view. (b) Left lateral profile view. (c) Right lateral profile view. (d) Inferior view.

**Figure 2:**
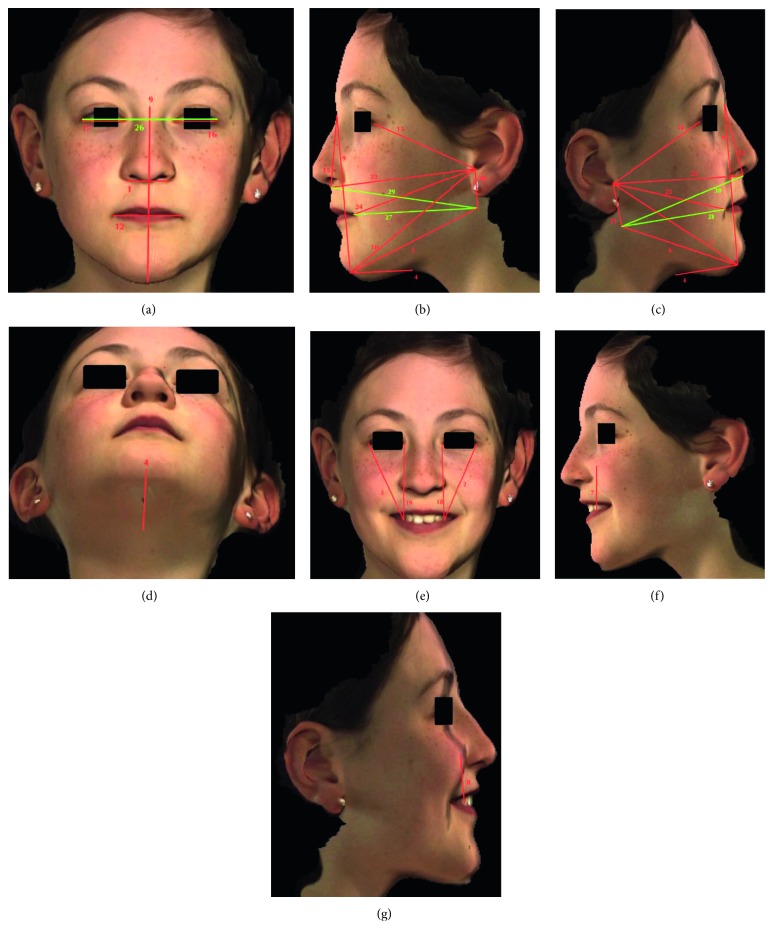
Frontal, left lateral profile, right lateral profile, inferior, frontal smiling, left lateral profile smiling, and right lateral profile smiling views of the facial scanner image, with numbered areas corresponding to landmarks. (a) Frontal View. (b) Left lateral profile view. (c) Right lateral profile view. (d) Inferior view. (e) Frontal view, smiling. (f) Left lateral profile view, smiling. (g) Right lateral profile view, smiling.

CBCTs and 3D facial scans from 10 patients out of the main sample were selected for reliability analysis (Figures 1 and 2). For the CBCT, reliability analysis was performed by initially obtaining 30 preselected distances (Table 1) based off of well-defined landmarks on soft and hard tissues (Table 2), for each of these 10 sets of patient images for both imaging modalities. All 30 distances were measured repeatedly, 3 times in total, for all 10 selected patient images for the CBCT and facial scan. A time span of one week took place after each of those three trials, in order to minimize any errors regarding the researcher's subjectivity of the placement of landmarks, especially those that were not too precise to locate on the images. Coordinates of the CBCT were analyzed for reliability calculations. For the facial scan, the same 30 distances (Table 1) were measured on 10 different facial scans, 3 times. Landmark distances were measured 3 times, leaving a week in between trials. Reliability calculations were performed from this data. Following landmark reliability calculations, the true data set of CBCT and facial scan images of the 30 selected patients were analyzed. Each of the 30 chosen distances was measured once on these patients for both imaging modalities. Descriptive statistics and paired *t*-test calculations were applied in order to obtain information regarding the relationships between distances on the skeletal and those on the facial tissue. The gold standard imaging modality is the CBCT, as it claims to have high precision (1 : 1 image to reality ratio), minimum deviation, and is highly reliable when evaluating linear distances for craniofacial analysis [4, 8–13].

## 3. Results

All measured distances presented excellent reliability, the lowest one being the left eye width of the facial scanner, with an intraclass correlation coefficient (ICC) of 0.847 (Tables 4 and 5). For CBCTs, the landmark with the highest mean error was 2.0 ± 1.6 mm on the *z*-axis for the spinal level landmark. The facial scanner's largest mean measurement error was 1.5 ± 0.9 mm for the distance of the left corner of the mouth to the left gonion.

When comparing the difference between facial scanner and CBCT measurements via the paired *t*-test, all data except that of the width between outer eye corners were statistically significant (*p* < 0.05). Although the *p* value of the width between outer eye corners is *p*=0.44, a very small facial scan to CBCT mean difference of 0.71 mm makes this measurement comparable.

Most measurements had a mean facial scan to CBCT difference of less than 9 mm. Such means indicate that distances measured on the CBCT and facial scan are very similar and thus comparable. However, measurements containing the left and right gonion, throat, corners of mouth, and subnasale had large facial scan to CBCT mean differences ranging from 16.66 mm (right corner of mouth to gonion) to 23.32 mm (left subnasale to gonion). Even though these means were relatively large, *paired* measurements with left and right sides had similar means. For example, the left gnathion to gonion measurement had a mean of 21.76 mm, while the right gnathion to gonion measurement had a mean of 20.79 mm, giving a difference in measurement of only 0.97 mm; although the mean is relatively large, both left and right sides are similar, indicating that they are comparable.

## 4. Discussion

Hard- and soft-tissue analyses are both critical tools for patient treatment planning and diagnosis and analysis of the patient over a long period of time. In contrary to conventional soft tissue and skeletal imaging tools such as patient photos and 2D analog films, 3D images of the patient are considered the ideal method of representing the face, and thus gives added information to the clinician, which in turn will give more realistic analyses [7]. Unlike using traditional 2D imaging to analyze 3D structures, which can have limited significance [7, 14, 15], comparing 3D hard to 3D soft tissue structures can be an improved alternate for the clinician to assess and evaluate cranial changes over time. Recently, several studies have adopted similar approaches in comparing 3D photography to CBCT concepts and have concluded that there is a close relationship between patient images taken by these two modalities.

In the present study, all measurements show a reasonable standard deviation between 2.57 mm (left eye width) to 9.91 mm (left gnathion to EAM). This shows that over a large sample size, these measurements are very similar, and less variable. However, the standard deviation for the gnathion to throat measurement is comparably large at 23.34 mm. This shows that there is lot of variation between the gnathion to throat measurement within a large sample.

The ratio between the soft tissue and CBCT measurements indicate their close correlation and any amount of variation or difference between them. The ratio percentages presented on Table 3 indicate the percentage of the CBCT distance measurements compared to that of the soft tissue measurements. Most ratio measurements are ±20%, but those that include the left and right gonion have a tendency to have smaller ratios, except for that of the left gonion to left EAM (+49.76%) and that of the right gonion to right EAM (+52.92%). These small ratios indicate that the CBCT, when measuring distances including the left and right gonion, tend to measure shorter than the soft tissue distances. A similar finding is seen in a study conducted by Naudi et al. [7], who evaluated the registration exactness of the simultaneous capture between a CBCT scan and a 3D surface of the face. Unlike the present study, the CBCT scans of this study captured soft-tissue measures to compare their superimposition with the 3D image capture. Naudi et al. concluded that in most of the facial surfaces, the level of superimposition in designated facial patches was 0.4 mm for simultaneous captures, denoting that superimpositions of the CBCT were smaller than those of the 3D image capture. The study also concluded that the most significant difference of superimposition between the CBCT and 3D image capture was in the chin area, with the mobile nature of the mandible being a large contributing factor of this result. It was mentioned that the relaxing atmosphere of the 3D image capture rooms may have led to patients slightly opening their jaws and bringing their teeth apart, leading to a slight increase in the degree of mouth opening and spatial changes of the related soft tissue. It can therefore be extrapolated that a larger degree of mouth opening of the soft tissue scans leads to a large superimposition, and thus, a larger difference compared to the CBCT image. These findings of Naudi et al. agree with the present study, as it was found that the tendency of losing measurement similarity, and thus having less of an intimate relationship between CBCT and facial scans was most prominent along the lateral portions of the face.

The tendency of having a lower correlation along the lateral portions of the face can be due to the variability of amounts of subcutaneous tissue present on each patient, but it may also be attributed to the increased amounts of larger, curved, boney surface areas on the lateral profile of the face. Toma et al. [16] indicated that due to the difficulty of placing points accurately on a patient's lateral profile, soft-tissue landmarks on both left and right lateral sides of the face are not highly reproducible. Such findings agree with the present study, since it was also found in this investigation that the left and right gonions have a tendency to elicit relatively large differences amongst soft tissue and CBCT landmark sites, whereas some of the smallest CBCT to soft tissue ratios were found along landmarks near the center of the face, including the width between outer eye corners (−0.74%) and the measurement between the nasion to gnathion (−4.38%).

Baumrind and Frantz [17] also found that the gonion was one of the least reliable landmarks to identify, whereas the nasion had a relatively smaller skeletal landmark estimating error. Although this study focused on 2D films, their findings can be extrapolated to 3D skeletal measurements on CBCT films. The study acknowledged that as a boney structure has a gradually curving edge, such as the gonion, the mean error of incorrect landmarking tends to be larger, leading to the large measurement error. Their findings agree with the present study, because in this investigation, landmark distances including either the left or right gonion were interpreted as those with the least amount of correlation between CBCT and 3D facial scanner (Table 3). The difficulty of locating the exact landmark of the left and right gonions may have lead to the large CBCT to soft tissue ratio difference, since landmarks may have been unintentionally placed along different areas of this largely curved boney edge. Additionally, despite the precise definition given to the gonion on the soft tissue (Table 1), the structure itself was found to be very challenging to visualize on facial images of the patient, as it is a structure that is easily hidden by subcutaneous tissue underneath.

As mentioned previously, measurements containing the left and right gonion, throat, corners of mouth, and subnasale had large facial scanner to CBCT mean differences. These measurements, along with others that had large means between facial scanner and CBCT data were those that had slightly different landmarks on the face between the two imaging modalities. Another study conducted by Maal et al. [18] had similar findings. This group investigated image fusion between soft tissue CBCT images and 3D photographs. It was found in their study that registration errors between CBCT and 3D images were largest at the lateral neck, mouth, and areas around the eyes. One of the causes of such dissimilarities was accredited to the fact of the inability of the CBCT to capture exact soft-tissue surfaces. Although this study focused on soft tissue comparison, the present study agrees with the concept that more registration error is found when there are different locations and definitions present for the same area on the face between two different imaging modalities. Soft-tissue CBCTs of Maal et al. were not of the same quality of comparison to that of the 3D images, and thus less precise locations would have been compared between these two imaging modalities. Similarly in this project, landmarks with a significant soft tissue to CBCT ratio > 20% are mainly due to the different definitions of the landmarks of the CBCT and soft tissue images, as defined in Table 1. The different definitions were created because it was acknowledged that hard- and soft-tissue landmarks are distinctly different in some definitions. For example, the large ratio percentage of the measurement of the width of the left eye (+24.21%) and the width of the right eye (+23.37%) raises mostly due to the different landmarking positions. The definition given for the “width of eye” is completely different: the CBCT defines this landmark as a distance between the frontozygomatic suture and the frontomaxillary suture, whereas the same landmark on the facial scanner is defined to be the distance from the lateral canthus to the medial canthus. Since both skeletal sutures extend beyond the lateral and medial canthi, this may be the cause of the CBCT's ratio being much higher. The larger discrepancy of the ratio due to different definitions is also seen in the “corners of mouth” landmark (−24.79%). The CBCT definition of the distance between the corners of the mouth is from one tip of the canine crown to the other, whereas the soft tissue definition is that of one cheilion to the other. Since individuals may have extended lip commissures, which potentially go further beyond the location of their canines, this may cause the CBCT to be seemingly shorter than that of the soft-tissue distances, even though this difference was due to a dissimilar definition.

Considering anterior-posterior (AP) and vertical measurements, left and right gonion landmarks were easily found on the CBCT but were difficult to locate on the facial scanner. Depending on the patient's size, the location of the gonion was easily or not easily found on the facial scanner. If not found, approximate landmarks were taken for the location of the gonion, which may have contributed to a larger difference in location compared to the CBCT. The measurement involving the throat (gnathion to throat, −10.99 mm) also had a relatively large facial scanner to CBCT mean difference, since slightly different landmarks were taken between the facial scanner and CBCT. On the facial scanner, the throat was defined and landmarked as the deepest part of the neck when viewed from the left and right sides. On the CBCT, the “throat” was landmarked as the spinal level that corresponded to the area of the throat, approximately at C2. Such different landmarks may have possibly contributed to a larger difference in the mean, with the CBCT measurement being larger than that of the facial scanner. Large mean differences of the throat may be related to low reproducibility of landmarks, since soft tissue anatomical features of the throat are much less clear than the hard tissue definition of this landmark. This may ultimately lead to low intraobserver reproducibility of this landmark [19].

Considering transverse measurements, the corners of the mouth also lead to a relatively large mean difference of 11.76 mm. On the facial scanner, the corner of each side of the mouth was landmarked to the furthest corner of the lips when the patient was not smiling. On the CBCT, the landmark for each corner of the mouth was taken as the canine for left and right sides. With some patients having a shifted or rotated canine, no canine, or orthodontic brackets, landmarking the canine on the CBCT had to be approximated, and for some cases, maxillary lateral incisors were used as landmarks instead. Distractions such as metal artefacts may reduce the exactness of the superimposition between the two imaging modalities [20]. The landmark for subnasale also had slightly different locations. On the facial scanner, the soft tissue subnasale point was used, which is the point of convergence of the nose and upper lip, directly beneath the nose. However on the CBCT, the central, most dense area directly under and between the two nasal sinuses was used for landmarking. Since the CBCT subnasale landmark was close to the anterior nasal spine, its measurements were more superior on the face compared to that of the facial scanner. A study conducted by Ayoub et al. [15] which investigated the superimposition of 3D data gathered from a CT scanner and a stereophotogrammetry tool found errors within an acceptable range of ±1.5 mm, with relatively large errors around the eyelid area. It was noted that the eyelid and eyebrow area is subject to surface shape differences when taken via these different imaging modalities, leading to this registration error. Additionally, Hwang et al. [19] stated that some anatomical structures such as the midlateral orbit do not clearly represent the actual anatomical structure of the soft tissues. The findings of both studies agree with the present study, as it was found that measurements containing outer and inner eyes generally had relatively large mean differences. The outer eye landmark on the facial scanner was defined as the most outer sharp part of the eye, and the inner eye landmark was also defined as the most inner sharp part seen on the eye. On the CBCT, the outer eye was defined as the suture between the frontal and zygomatic bones, and the inner eye landmark was located and the suture between the frontal bone and maxilla, near the nasal bone. Since these sutures are more superior on the face than the actual soft tissue outer and inner eye corners, a slightly different mean between the facial scanner and CBCT can be seen; CBCT values are slightly larger, and thus mean difference measurements for left canine to outer eye (−8.55 mm), right canine to outer eye (−7.43 mm), left eye width (−7.49 mm), right eye width (−7.29 mm), left inner eye to canine (−7.25 mm), and right inner eye to canine (−6.81 mm) are negative. As such, these findings indicate that soft- and hard-tissue landmarks of the eye are difficult to reproduce.

Nahm et al. [21] also similarly found the registration relationship between CBCT and facial surfaces to be very close and concluded that merging CBCT and facial scans can produce a much truthful image of the patient to give the orthodontist enhanced diagnostic information and lessen errors in diagnosis. These findings agree with the present study, since it was found that other than some of the few distances mentioned above, many other ratios have excellent soft tissue to CBCT ratio percentages, such as the width between outer eye corners (−0.74%), left (−2.94%) and right (−3.99%) corners of the mouth to EAM, and the nasion to gnathion (−4.38%) measurement. This indicates that the CBCT has a tendency to superimpose very closely to distances of the facial scanner.

Limitations to this study exist which warrant changes to be made for further improvement of this study. Due to the data being collected in a retrospective fashion, there was no method in which head positions for the facial scan and CBCT could have been strictly controlled. Patients were only advised to keep their head in the natural position, with their Frankfort plane parallel to the floor. The result of such minor head position changes of each patient may lead to changes in the position of mobile facial structures, such as the mandible. This will lead to slight changes in landmark positioning of areas such as the gonion, causing a greater difference between the two imaging modalities.

The fact that this study was based off of retrospective data also serves as a limitation in that it limited the sample size. A high-enough sample size is needed based on *a priori* calculations, but such calculations were not performed, since there were only a few patient files within the database that included both facial scans and full field of view CBCTs. Thus, within the small given number of available data to work with, the number 30, was chosen, which was the highest number based on availability of data.

In this study, the gold standard was considered to be the CBCT. This assumption was made based on multiple, high-quality research papers and articles [4, 8–13]. Although it is an educated assumption, this assumption serves as a limitation to this study. In order to improve clinical precision, it is important to complete real distance measurements on the patient's face and compare them very carefully with both digital methods.

Most measurements have a reasonably small facial scanner to CBCT mean difference. Even if the difference is relatively large, this can be explained by knowing that some landmarks were slightly different in terms of location on the CBCT and facial scanner. Additionally, even if the means and standard deviations may be large, all paired measurements with left and right sides have similar values within at most 2.35 mm from each other, indicating that such measurements are still comparable.

## 5. Conclusion

The mean soft tissue to CBCT facial distances tend to be within ±20%, with a tendency for the facial scan measurements to be slightly larger than CBCT equivalents. In general, correlation between the facial scan and CBCT tends to be smaller at lateral and mobile areas of the face such as the gonion. Right and left gonions were areas of the face with a high level of difference between landmark sites of the two imaging modalities. There is a general tendency of obtaining less correlation on boney structures as they increase in dimensional size and increase their curvature. Other significant measurement dissimilarities were due to differences in landmark definition between CBCT and facial scan, in areas such as the throat, corners of mouth, and outer/inner eyes. Areas of the face that have a tendency to have high differences between surface shapes, such as the eyelid and eyebrow region, had relatively low correlation between their soft tissues and corresponding hard-tissue landmarks. Some of the limitations of this study, which may lead to lower correlation in some facial landmarks, may include data from a retrospective database, nonspecific head positions of the patient, as well as an indefinite gold standard imaging modality.

## Figures and Tables

**Table 1:** Measured and defined distances depending on the image used.

	Landmarks	Description of distances on CBCT	Description of distances on facial scanner
1	Width of nose	Left bottom-most skeletal corner under the nasal aperture to the right bottom-most skeletal corner	Left alar curvature point (the most lateral part of the curved base of the ala) to the right alar curvature point
2	Left canine to left outer eye	Most tip of left canine crown to left frontozygomatic suture	Most tip of left canine (patient smiling) to left lateral canthus
3	Right canine to right outer eye	Most tip of right canine crown to right frontozygomatic suture	Most tip of right canine (patient smiling) to right lateral canthus
4	Gnathion to throat	Lowest point of the midline of the mandible to C3-C4 cervical vertebrae	Lowest point of the midline of the mandible to the most indented location of the throat between the chin and neck
5	Gnathion to left gonion	Lowest point of the mandibular midline to the lowest, most posterior, and lateral point of the left mandibular angle	Lowest point of the mandibular midline to the lowest, most posterior, and lateral point of the left mandibular angle
6	Gnathion to right gonion	Lowest point of the mandibular midline to the lowest, most posterior, and lateral point of the right mandibular angle	Lowest point of the mandibular midline to the lowest, most posterior, and lateral point of the right mandibular angle
7	Left canine to left cheekbone	Most tip of left canine crown to most prominent frontal portion of the left zygomatic bone	Most tip of left canine crown (patient smiling) to most prominently raised point of left cheek area, most likely an area under the left lateral canthus
8	Right canine to right cheekbone	Most tip of right canine crown to most prominent frontal portion of the right zygomatic bone	Most tip of right canine crown (patient smiling) to most prominently raised point of right cheek area, most likely an area under the right lateral canthus
9	Nasion to gnathion	Distinctly depressed area between the intersection of the frontal bone and two nasal bones to the lowest point of the mandibular midline.	Distinctly depressed area directly between the eyes and superior to the bridge of the nose to the lowest point of the mandibular midline
10	Gnathion to left external auditory meatus (EAM)	Lowest portion of the mandibular midline to lowest bony portion of the left hollow canal of the tympanic portion of the temporal bone, posterior to the condylar process of the mandible	Lowest portion of the mandibular midline to the left lowest portion of the hollow ear canal
11	Gnathion to right EAM	Lowest portion of the mandibular midline to lowest bony portion of the right hollow canal of the tympanic portion of the temporal bone, posterior to the condylar process of the mandible	Lowest portion of the mandibular midline to the right lowest portion of the hollow ear canal
12	Corners of mouth	Tip of left canine crown to tip of right canine crown	Left cheilion (left labial commissure) to right cheilion (right labial commissure)
13	Left EAM to left outer eye corner	Lowest bony portion of the left hollow canal of the tympanic portion of the temporal bone, posterior to the condylar process of the mandible, to the left lateral canthus of eye	Left lowest portion of the hollow ear canal to the left lateral canthus of eye
14	Right EAM to right outer eye corner	Lowest bony portion of the right hollow canal of the tympanic portion of the temporal bone, posterior to the condylar process of the mandible, to the right lateral canthus of eye	Right lowest portion of the hollow ear canal to the right lateral canthus of eye
15	Bottom of nose to nasion	Anterior nasal spine to the distinctly depressed area between the intersection of the frontal bone and two nasal bones	Subnasale (the midpoint of the angle at the nasal base where the lower border of the nasal septum and the upper lip surface meets) to the distinctly depressed area directly between the eyes and superior to the bridge of the nose
16	Width of left eye	Left frontozygomatic suture to left frontomaxillary suture	Left lateral canthus to left medial canthus
17	Width of right eye	Right frontozygomatic suture to right frontomaxillary suture	Right lateral canthus to right medial canthus
18	Left inner eye to left canine	Left frontomaxillary suture to tip of left canine crown	Left medial canthus to tip of left canine crown (patient smiling)
19	Right inner eye to right canine	Right frontomaxillary suture to tip of right canine crown	Right medial canthus to tip of right canine crown (patient smiling)
20	Left gonion to left EAM	Lowest, most posterior, and lateral point of the left mandibular angle to the lowest bony portion of the left hollow canal of the tympanic portion of the temporal bone, posterior to the condylar process of the mandible	Most posterior and lateral point of the left mandibular angle to the left most lowest portion of the hollow ear canal opening
21	Right gonion to right EAM	Lowest, most posterior, and lateral point of the right mandibular angle to the lowest bony portion of the right hollow canal of the tympanic portion of the temporal bone, posterior to the condylar process of the mandible	Most posterior and lateral point of the right mandibular angle to the right most lowest portion of the hollow ear canal opening
22	Bottom of nose to left EAM	Anterior nasal spine to the lowest bony portion of the left hollow canal of the tympanic portion of the temporal bone, posterior to the condylar process of the mandible	Subnasale (the midpoint of the angle at the nasal base where the lower border of the nasal septum and the upper lip surface meets) to the left most lowest portion of the hollow ear canal opening
23	Bottom of nose to right EAM	Anterior nasal spine to the lowest bony portion of the right hollow canal of the tympanic portion of the temporal bone, posterior to the condylar process of the mandible	Subnasale (the midpoint of the angle at the nasal base where the lower border of the nasal septum and the upper lip surface meets) to the right most lowest portion of the hollow ear canal opening
24	Left corner of mouth to left EAM	Tip of left canine crown to the lowest bony portion of the left hollow canal of the tympanic portion of the temporal bone, posterior to the condylar process of the mandible	Left cheilion (left labial commissure) to the left most lowest portion of the hollow ear canal opening
25	Right corner of mouth to right EAM	Tip of right canine crown to the lowest bony portion of the right hollow canal of the tympanic portion of the temporal bone, posterior to the condylar process of the mandible	Right cheilion (right labial commissure) to the right most lowest portion of the hollow ear canal opening
26	Width between outer eye corners	Left frontozygomatic suture to right frontozygomatic suture	Left lateral canthus to right lateral canthus
27	Left corner of mouth to left gonion	Tip of left canine crown to the lowest, most posterior, and lateral point of the left mandibular angle	Left cheilion (left labial commissure) to the most posterior and lateral point of the left mandibular angle
28	Right corner of mouth to right gonion	Tip of right canine crown to the lowest, most posterior, and lateral point of the right mandibular angle	Right cheilion (right labial commissure) to the most posterior and lateral point of the right mandibular angle
29	Bottom of nose to left gonion	Anterior nasal spine to lowest, most posterior, and lateral point of the left mandibular angle	Subnasale (the midpoint of the angle at the nasal base where the lower border of the nasal septum and the upper lip surface meets) to most posterior and lateral point of the left mandibular angle
30	Bottom of nose to right gonion	Anterior nasal spine to lowest, most posterior, and lateral point of the right mandibular angle	Subnasale (the midpoint of the angle at the nasal base where the lower border of the nasal septum and the upper lip surface meets), to most posterior and lateral point of the right mandibular angle

**Table 2:** Definition of landmarks used for measuring specific distances, depending on the imaging modality used.

	Landmark	Description of landmark on CBCT	Description of landmark on soft tissue
1	Sides of nose	Left/right bottom most skeletal corner under the nasal aperture	Left/right alar curvature (most lateral part of the curved base of the ala)
2	Canine	Most tip of the left/right canine crown	Most tip of the left/right canine crown
3	Outer eye	Left/right frontozygomatic suture	Left/right lateral canthus
4	Gnathion	Lowest point of the midline of the mandible	Lowest point of the midline of the mandible
5	Throat	C3-C4 cervical vertebrae location	Most indented location of the throat between the chin and neck
6	Gonion	Most posterior and lateral point of the left/right mandibular angle	Most posterior and lateral point of the left/right mandibular angle on the skin
7	Cheekbone	Most prominent frontal portion of the left/right zygomatic bone	Most prominently raised point of the left/right cheek area, most likely an area under the left/right lateral canthus
8	Nasion	Distinctly depressed area between the intersection of the frontal bone and two nasal bones	Distinctly depressed area directly between the eyes and superior to the bridge of the nose
9	External auditory meatus (EAM)	Lowest bony portion of the left/right hollow canal of the tympanic portion of the temporal bone, posterior to the condylar processes of the mandible	Left/right lowest portion of the hollow ear canal
10	Corners of mouth	Tip of left/right canine crowns	Left/right cheilion (left/right labial commissures)
11	Bottom of nose	Anterior nasal spine	Subnasale (midpoint of the angle at the nasal base where the lower border of the nasal septum and upper lip surface meets)
12	Inner eye	Left/right frontomaxillary suture	Left/right medial canthus

**Table 3:** Statistics of soft tissue distances and their CBCT equivalents, including average mean measurements, standard deviation, the difference between the average mean measurements, and the ratio of the CBCT distances to the soft-tissue distances in percentage format, *p* values, and 95% confidence intervals of the differences between the facial scan and CBCT.

	Landmarks	Soft tissue measurements		CBCT hard tissue measurements		*p* value	95% confidence interval of the differences (facial scan-CBCT)		Facial scanner and CBCT mean difference (mm)	Ratio of soft tissue and CBCT distances (%)
		Average mean (mm)	Standard deviation	Average mean (mm)	Standard deviation		Lower	Upper		
1	Width of nose	25.26	2.53	21.65	3.33	0.001	−15.04	9.43	3.61	−14.29
2	Left canine to left outer eye	70.05	3.97	78.60	5.62	0.001	−17.70	−5.79	−8.55	+12.21
3	Right canine to right outer eye	70.47	4.49	77.90	5.33	0.001	−9.17	−5.68	−7.43	+10.54
4	Gnathion to throat	68.68	24.07	79.67	10.09	0.019	−20.88	−0.42	−10.99	+16.00
5	Gnathion to left gonion	101.68	8.37	79.92	7.72	0.001	21.59	31.86	21.76	−21.40
6	Gnathion to right gonion	100.56	8.33	79.77	6.03	0.001	21.11	31.55	20.79	−20.67
7	Left canine to left cheekbone	48.18	3.28	43.51	3.62	0.001	−7.04	8.58	4.67	−9.69
8	Right canine to right cheekbone	49.09	4.07	42.07	6.32	0.001	−20.36	16.31	7.02	−14.30
9	Nasion to gnathion	116.51	8.51	111.41	8.58	0.003	0.18	14.99	5.10	−4.38
10	Gnathion to left external auditory meatus (EAM)	128.58	12.71	120.86	9.25	0.001	−4.89	15.28	7.72	−6.00
11	Gnathion to right EAM	127.69	12.15	120.30	9.25	0.001	3.47	15.03	7.39	−5.79
12	Corners of mouth	47.43	4.80	35.67	3.74	0.001	−6.24	17.90	11.76	−24.79
13	Left EAM to left corner eye	80.71	5.52	72.88	5.10	0.001	0.29	10.71	7.83	−9.70
14	Right EAM to right corner eye	80.80	5.59	72.87	5.00	0.001	−1.95	10.40	7.93	−9.81
15	Bottom of nose to nasion	47.71	4.32	54.05	6.19	0.001	−8.87	−4.29	−6.34	+13.29
16	Width of left eye	30.94	2.28	38.43	2.79	0.001	−26.87	−1.01	−7.49	+24.21
17	Width of right eye	31.19	2.38	38.48	2.62	0.001	−8.30	−6.27	−7.29	+23.37
18	Left inner eye to left canine	63.27	4.46	70.51	5.48	0.001	−20.34	−2.78	−7.24	+11.44
19	Right inner eye to right canine	63.63	4.63	70.44	5.72	0.001	−43.33	5.63	−6.81	+10.70
20	Left gonion to left EAM	35.25	5.90	52.79	6.53	0.001	−26.02	−14.41	−17.54	+49.76
21	Right gonion to right EAM	34.88	5.17	53.34	7.38	0.001	−27.41	−14.06	−18.46	+52.92
22	Bottom of nose to left EAM	119.07	8.82	101.26	9.21	0.001	14.00	24.11	17.81	−14.96
23	Bottom of nose to right EAM	120.17	8.80	101.29	8.81	0.001	−11.12	30.86	18.88	−15.71
24	Left corner of mouth to left EAM	98.68	7.96	95.78	7.54	0.015	−6.78	6.25	2.90	−2.94
25	Right corner of mouth to right EAM	98.86	8.03	94.92	7.70	0.001	−0.19	7.08	3.94	−3.99
26	Width between outer eye corners	94.88	5.42	94.18	11.69	0.437	−7.03	10.41	0.70	−0.74
27	Left corner of mouth to left gonion	85.72	6.76	68.43	8.44	0.001	7.96	23.08	17.29	−20.17
28	Right corner of mouth to right gonion	84.42	7.15	67.76	6.11	0.001	15.41	20.23	16.66	−19.73
29	Bottom of nose to left gonion	109.42	8.33	86.10	9.35	0.001	21.29	32.56	23.32	−21.31
30	Bottom of nose to right gonion	110.41	8.98	87.26	8.93	0.001	19.84	33.63	23.15	−20.97

**Table 4:** Intraclass correlation coefficients (ICC) of facial scan landmarks, lower and upper limits of their 95% confidence intervals, and their corresponding *p* values.

	Landmarks	Intraclass correlation coefficient	95% confidence intervals of soft tissue ICC		*p* value
			Lower	Upper	
1	Width of nose	0.960	0.883	0.989	0.001
2	Left canine to left outer eye	0.965	0.900	0.991	0.001
3	Right canine to right outer eye	0.990	0.970	0.997	0.001
4	Gnathion to throat	0.999	0.997	1.000	0.001
5	Gnathion to left gonion	0.999	0.998	1.000	0.001
6	Gnathion to right gonion	0.999	0.998	1.000	0.001
7	Left canine to left cheekbone	0.947	0.846	0.986	0.001
8	Right canine to right cheekbone	0.971	0.918	0.992	0.001
9	Nasion to gnathion	1.000	0.999	1.000	0.001
10	Gnathion to left external auditory meatus (EAM)	1.000	0.999	1.000	0.001
11	Gnathion to right EAM	1.000	0.999	1.000	0.001
12	Corners of mouth	0.974	0.925	0.993	0.001
13	Left EAM to left corner eye	0.971	0.914	0.992	0.001
14	Right EAM to right corner eye	0.972	0.921	0.992	0.001
15	Bottom of nose to nasion	0.989	0.968	0.997	0.001
16	Width of left eye	0.847	0.539	0.959	0.001
17	Width of right eye	0.875	0.643	0.966	0.001
18	Left inner eye to left canine	0.993	0.979	0.998	0.001
19	Right inner eye to right canine	0.993	0.980	0.998	0.001
20	Left gonion to left EAM	0.994	0.983	0.998	0.001
21	Right gonion to right EAM	0.991	0.974	0.998	0.001
22	Bottom of nose to left EAM	0.998	0.994	0.999	0.001
23	Bottom of nose to right EAM	0.996	0.990	0.999	0.001
24	Left corner of mouth to left EAM	0.996	0.990	0.999	0.001
25	Right corner of mouth to right EAM	0.996	0.989	0.999	0.001
26	Width between outer eye corners	0.989	0.968	0.997	0.001
27	Left corner of mouth to left gonion	0.982	0.950	0.995	0.001
28	Right corner of mouth to right gonion	0.992	0.978	0.998	0.001
29	Bottom of nose to left gonion	0.994	0.982	0.998	0.001
30	Bottom of nose to right gonion	0.991	0.975	0.998	0.001

**Table 5:** Intraclass correlation coefficients (ICC) of CBCT landmarks, lower and upper limits of their 95% confidence intervals, and their corresponding *p* values.

	Landmark	Intraclass correlation coefficient	95% confidence intervals of CBCT ICC		*p* value
			Lower	Upper	
1	Left nose radiolucency	0.990	0.971	0.997	0.001
2	Right nose radiolucency	0.967	0.908	0.991	0.001
3	Left canine	0.993	0.980	0.998	0.001
4	Right canine	0.997	0.989	0.999	0.001
5	Gnathion	0.979	0.940	0.994	0.001
6	Throat (spinal level)	0.987	0.964	0.997	0.001
7	Left gonion	0.999	0.997	1.000	0.001
8	Right gonion	0.998	0.995	1.000	0.001
9	Left zygomatic process	0.975	0.927	0.993	0.001
10	Right zygomatic process	0.969	0.910	0.991	0.001
11	Left external auditory meatus	0.998	0.995	1.000	0.001
12	Right external auditory meatus	0.992	0.977	0.998	0.001
13	Left frontozygomatic suture (outer corner of eye)	0.987	0.963	0.997	0.001
14	Right frontozygomatic suture (outer corner of eye)	0.988	0.967	0.997	0.001
15	Subnasale	0.992	0.978	0.998	0.001
16	Nasion	0.991	0.973	0.997	0.001
17	Left frontonasal suture (inner corner of eye)	0.962	0.891	0.990	0.001
18	Right frontonasal suture (inner corner of eye)	0.977	0.934	0.994	0.001

## Data Availability

The data used to support the findings of this study are available from the corresponding author upon request.
